# Dairy and cardiovascular health: Friend or foe?

**DOI:** 10.1111/nbu.12086

**Published:** 2014-05-19

**Authors:** O Markey, D Vasilopoulou, D I Givens, J A Lovegrove

**Affiliations:** * Hugh Sinclair Unit of Human Nutrition and Institute for Cardiovascular and Metabolic Research (ICMR), Department of Food and Nutritional Sciences, University of ReadingUK; † Food Production and Quality Research Division, School of Agriculture, Policy and Development, Faculty of Life Sciences, University of ReadingUK

**Keywords:** arterial stiffness, blood pressure, cardiovascular disease, dairy products, milk, saturated fatty acids

## Abstract

Cardiovascular disease (CVD) prevalence at a global level is predicted to increase substantially over the next decade due to the increasing ageing population and incidence of obesity. Hence, there is an urgent requirement to focus on modifiable contributors to CVD risk, including a high dietary intake of saturated fatty acids (SFA). As an important source of SFA in the UK diet, milk and dairy products are often targeted for SFA reduction. The current paper acknowledges that milk is a complex food and that simply focusing on the link between SFA and CVD risk overlooks the other beneficial nutrients of dairy foods. The body of existing prospective evidence exploring the impact of milk and dairy consumption on risk factors for CVD is reviewed. The current paper highlights that high milk consumption may be beneficial to cardiovascular health, while illustrating that the evidence is less clear for cheese and butter intake. The option of manipulating the fatty acid profile of ruminant milk is discussed as a potential dietary strategy for lowering SFA intake at a population level. The review highlights that there is a necessity to perform more well-controlled human intervention-based research that provides a more holistic evaluation of fat-reduced and fat-modified dairy consumption on CVD risk factors including vascular function, arterial stiffness, postprandial lipaemia and markers of inflammation. Additionally, further research is required to investigate the impact of different dairy products and the effect of the specific food matrix on CVD development.

## Introduction

Although mortality from cardiovascular disease (CVD) is now falling in most European countries, CVD is ranked as the leading cause of mortality in the UK and worldwide (BHF 2010; WHO 2011; Nichols *et al*. 2012). It is envisaged that the global prevalence of CVD will continue to increase and is expected to be responsible for more than 23.6 million deaths by 2030 (Smith *et al*. 2012), a figure that is largely attributable to today's dramatic demographic changes with increasing proportions of ageing and obese groups. The outcome of today's obesity and ageing trends may, if not moderated, result in unsustainable costs to global society. Currently, CVD costs the European Union (EU) economy approximately €196 billion per annum in direct and indirect charges, with the cost per person in the UK exceeding the EU average (Nichols *et al*. 2012). There is growing pressure to reduce risk factors for CVD at a population level. An atherogenic diet, characterised by a high intake of dietary saturated fatty acids (SFA), is a key modifiable risk factor for CVD. While the effects of the amount and type of dietary fat have been examined in relation to CVD, less focus has been placed on the role of animal-derived staple foods such as milk and dairy products, which are significant dietary sources of SFA.

## Saturated fat consumption

In order to reduce the population health burden of CVD, a number of strategies can be addressed; these include increasing physical activity, improving weight profiles and reducing tobacco and alcohol intake. However, this paper will focus on strategies for combatting the overconsumption of dietary SFA.

The adverse effect of SFA on CVD risk is well established; this is primarily mediated via increases in serum lipids, particularly low-density lipoprotein (LDL)-cholesterol (Mensink *et al*. 2003). Current dietary recommendations include an intake of dietary SFA of less than 10% of total energy (DH 1991; WHO 2008). The current rolling *National Diet and Nutrition Survey* (*NDNS*), as outlined in Table 1, highlights that this dietary target is exceeded by the majority of men, women and children in the UK (DH 2012).

**Table 1:** Average intake of saturated fatty acids in absolute terms and as a percentage of food/total energy, by age and gender (NDNS 2008/2009–2010/2011)

	Boys/Men			Girls/Women		
		
	4–18 years	19–64 years	≥65 years	4–18 years	19–64 years	≥65 years
SFA (g/day)	25.8 ± 8.6	28.8 ± 12.1	29.3 ± 10.7	22.7 ± 8.1	22.0 ± 9.8	23.2 ± 9.0
% food energy	12.9 ± 2.6	12.8 ± 3.3	14.4 ± 3.5	12.9 ± 2.7	12.6 ± 3.4	14.0 ± 3.7
% total energy	12.9 ± 2.6	12.0 ± 3.4	13.6 ± 3.4	12.9 ± 2.8	12.0 ± 3.3	13.7 ± 3.3

Source: DH (2012).

Mean ± SD. NDNS, National Diet and Nutrition Survey; SFA, saturated fatty acids.

### Substitution of dietary saturated fat with carbohydrate or unsaturated fatty acids

The question of whether replacement of dietary SFA with carbohydrate (CHO), *cis-*polyunsaturated fatty acids (*cis*-PUFA) or *cis*-monounsaturated fatty acids (*cis*-MUFA) can have beneficial effects on CVD mortality and risk has received considerable attention (Astrup *et al*. 2011; Hooper *et al*. 2012; Vafeiadou *et al*. 2012). There would seem to be no clear evidence for a benefit of substituting CHO for SFA in the prevention of CVD (Astrup *et al*. 2011; Hooper *et al*. 2012) but there is some recent evidence for the benefit of replacing SFA with unsaturated fatty acids (Jakobsen *et al*. 2009; Micha & Mozaffarian 2010; Hooper *et al*. 2012). There is good evidence that replacing SFA with *cis*-PUFA will reduce CVD mortality and risk markers, although there is less information on the effects of replacing SFA with *cis*-MUFA, mainly because few relevant randomised controlled trials have been performed (Mozaffarian *et al*. 2010). Overall, the Cochrane meta-analysis of Hooper *et al*. (2012) identified that a reduction of dietary SFA intake (where it is replaced by unsaturated fat) and/or reduction of total dietary fat lowered the risk of cardiovascular events by 14% [relative risk (RR) 0.86; 95% confidence interval (CI) 0.77–0.96] but had no effect on total mortality.

## The contribution of dairy products to saturated fat intake

Milk and dairy products are one of the most significant contributors to SFA intake in the UK diet. In the context of this review, we are defining dairy as cows' milk or any food product derived from cows' milk. However, it should be noted that some dairy products (including butter and cream) do not fall into the ‘milk and dairy’ section of the *eatwell* plate because they are predominantly viewed as significant sources of fat (FSA 2011). Recent *NDNS* data (Table 2) suggest that milk and dairy products are responsible for 22–25% of SFA consumption in adults (DH 2011); however, it should be noted that this analysis failed to take into account the intake of composite dairy dishes (*e.g.* pizza and lasagne) as well as butter consumption, the latter contributing around 5% to daily average saturated fat intakes; thus SFA intake from milk and dairy product consumption may be underestimated.

**Table 2:** Percentage contribution of food groups to average daily total saturated fatty acid intake, by age and gender (NDNS 2008/2009–2009/10)

	Boys/Men			Girls/Women		
		
Food groups (%)	4–18 years	19–64 years	≥65 years	4–18 years	19–64 years	≥65 years
Cereals and cereal products	25	18	17	24	20	18
Milk and milk products	25	22	28	27	22	24
Eggs and egg dishes	2	3	3	2	4	4
Fat spreads	9	10	13	8	10	16
Meat and meat products	22	27	22	20	23	19
Fish and fish dishes	2	3	4	1	3	5
Vegetables and potatoes	5	7	5	6	7	6
Savoury snacks	2	1	0	2	1	0
Nuts/seeds/fruits	0	1	1	0	2	0
Sugars/preserves/confectionary	7	4	2	7	5	3
Drinks	0	1	1	0	1	0
Miscellaneous	2	3	3	2	4	3

Source: DH (2011).

NDNS, National Diet and Nutrition Survey.

### Effects of saturated fat in dairy products on plasma lipids and lipoproteins

A detailed review by Huth and Park (2012) revealed that diets higher in SFA from whole milk and butter increase LDL-cholesterol but may also have a beneficial impact on high-density lipoprotein (HDL)-cholesterol concentrations, resulting in a neutral or beneficial effect on the total cholesterol:HDL-cholesterol ratio. Fermented dairy products including cheese and yogurt appear to have a different impact on circulating levels of plasma lipid and lipoproteins. For example, compared to butter matched for milk fat intake, consumption of hard cheese for a 6-week period led to significantly lower concentrations of total cholesterol, LDL-cholesterol and HDL-cholesterol (Hjerpsted *et al*. 2011). However, it should be noted that butter is not necessarily a good comparator. There are a number of confounding variables that negate the drawing of firm conclusions in relation to cheese and CVD risk due to the differential effect of individual cheese varieties that differ in macronutrient content, degree of fermentation and food matrix (Huth & Park 2012). Furthermore, it highlights that the impact of dairy products on CVD risk factors may be dependent on the specific dairy food, even when supplying the same mass of dairy fat. However, further long-term studies are necessary before firmer conclusions can be drawn on the relative impact of milk and milk-derived food consumption on plasma lipids and lipoproteins.

## The consumption of milk and milk-derived products and cardiovascular disease

### Potential benefits of milk and dairy consumption

Dairy fats are high in SFA; however, ruminant milk is a complex food and there is much debate as to whether nutrients within dairy foods act independently or synergistically in relation to chronic disease development. The association of SFA with CVD development may be dependent on other nutrients/macronutrients in the matrix of the SFA-containing food (de Oliveira Otto *et al*. 2012). Milk is a significant source of a number of essential micronutrients including calcium, potassium and iodine. Calcium, for example, has a higher bioavailability in milk compared with that present in some other foods (Weaver *et al*. 1999). Furthermore, mineral bioavailability is enhanced by the lack of inhibitors present in milk, including phytates and oxalates, and by the presence of lactose and certain amino acids that may promote mineral absorption (FAO 2013). Moreover, lipids mediate the delivery of essential fat-soluble vitamins, including vitamins D and A, and fatty acids associated with dairy products, namely conjugated linoleic acids, may also have cardio-protective properties, although the data are inconsistent and require confirmation in further human studies (Dilzer & Park 2012). Furthermore, emerging evidence suggests that plasma phospholipid *trans*-palmitoleic acid (*trans* 16:1 *n*-7), a circulating fatty acid biomarker positively correlated with self-reported intakes of dairy fat intake (whole-fat dairy products and butter), is associated with a more favourable metabolic profile and incident diabetes rate (Micha & Mozaffarian 2010; Mozaffarian *et al*. 2013). However, the aforementioned findings regarding *trans* 16:1 *n*-7 should be interpreted with caution as they do not necessarily prove cause and effect; thus, oversimplifying the relationship between dairy product intake (in terms of SFA content) and CVD risk may be misleading (Givens 2012).

### Changing trends in dairy consumption

UK trends in dairy product consumption have changed markedly over recent years; this could be partly due to the negative connotations surrounding dairy products and SFA content. *Family Food* statistics published by the Department for Environment, Food and Rural Affairs (Defra) over the past 20 years show a decline in total liquid milk consumption, largely brought about by a decrease in whole milk consumption, which now accounts for around 20% of milk consumed in the UK today. Butter consumption has decreased markedly from the 1970s by about 70%, and fell around 2% lower in the decade between 2001 and 2011, while cheese and yogurt consumption increased by around 5% and 30%, respectively, over the same time period (AHDB-DairyCo 2012).

### Dietary patterns associated with milk and dairy product consumption

Analysis of dietary patterns is recognised as an alternate and complementary strategy for investigating the association between diet and disease risk. As dietary patterns are more representative of overall food consumption, they may facilitate a more valid prediction of CVD risk compared with assessment of an individual nutrient or food (Hu 2002). Prospective data from 88 517 middle-aged women indicated that adherence to the Dietary Approaches to Stop Hypertension (DASH) diet, characterised by a moderate intake of low-fat dairy, legumes and nut products, high intake of fruit, vegetables and wholegrains, and low intake of red meat, was inversely associated with risk of coronary heart disease (CHD) and stroke during a 24-year follow-up period (Fung *et al*. 2008). This finding was in agreement with the *Atherosclerosis Risk in Communities Study* which illustrated that a dietary pattern rich in dairy and nut products, but less meat, is associated with a lower risk of incident hypertension in middle-aged adults (Weng *et al*. 2013). However, many international and national dietary guidelines recommend the reduced intake of full-fat dairy products as one aspect of a dietary pattern linked to reducing risk of CHD (Erlinger & Appel 2005).

### Dairy consumption and cardiovascular disease risk: Prospective evidence

A number of studies have investigated the effect of milk and dairy products on different CVD events. As previously mentioned, there is a lack of robust evidence on the potential differential effects of individual dairy foods on CVD risk as most observational studies combine dairy products as a single food group, although milk is better studied. A meta-analysis of prospective cohort studies reported that, overall, high milk consumption does not increase the RR of CHD (Elwood *et al*. 2008). A second meta-analysis, which combined prospective cohort and clinical studies, revealed that there was insufficient evidence for an association between milk consumption and CHD (RR 0.94; 95% CI 0.75–1.13) (Mente *et al*. 2009). A later more extensive meta-analysis on milk and dairy consumption and CVD events concluded that high consumption of milk was related to a significant reduction in risk of stroke development (Elwood *et al*. 2010). Table 3 is largely based on Elwood *et al*. (2010) but has been updated by the addition of data from six recently published studies (Bonthuis *et al*. 2010; Goldbohm *et al*. 2011; Sonestedt *et al*. 2011; Soedamah-Muthu *et al*. 2012; Avalos *et al*. 2013; van Aerde *et al*. 2013). A recent study showed that compared with the lowest quintile of dairy consumption, total dairy intake was inversely related to myocardial infarction (MI) risk following an 11.6-year follow-up period (HR 0.77; 95% CI 0.63–0.95). Further analysis revealed that butter used on bread was positively correlated with MI risk, while total cheese intake had an inverse risk association (Patterson *et al*. 2013). This evidence supports the hypothesis that dairy products, excluding butter, are associated with no detrimental effect, and in some cases a significant reduced CVD risk.

**Table 3:** Summary of the relative risk for milk and dairy consumption and CVD events

Disease outcome	Number of cohort studies (number used in analyses)	Adjusted RR (95% CI) for milk/dairy consumption*	Significance of heterogeneity between studies
Ischaemic heart disease	22 (17)	0.92 (0.86, 0.99)	*P* = 0.765
All strokes	12	0.79 (0.68, 0.91)	*P* = 0.001
Haemorrhagic stroke	5	0.75 (0.60, 0.94)	*P* = 0.014
Subarachnoid bleed	3	0.93 (0.84, 1.02)	*P* = 0.004
All-cause mortality	12 (9)	0.91 (0.78, 1.05)	*P* = 0.070

Source: Adapted from Givens *et al*. (2014).

* Estimate of the risk of each disease in individuals with the highest consumption of milk/dairy products compared to the risk in individuals with the lowest consumption.

CI, confidence interval; CVD, cardiovascular disease; RR, relative risk.

Hypertension is one of the key risk factors for CVD development and is influenced by gene polymorphisms, nutrition, the environment and interactions between these factors. Milk and milk-derived products provide essential micronutrients (such as calcium, potassium and iodine) and protein (whey, casein and specific bioactive peptides), some of which have been associated with beneficial hypotensive effects, either independently or synergistically (Kris-Etherton *et al*. 2009). There are a number of proposed mechanisms by which milk and its components could reduce blood pressure (BP; for a detailed review, see Fekete *et al*. 2013). Bioactive peptides present in casein and whey proteins have been observed to play a role in controlling BP by inhibiting the action of angiotensin-I-converting enzyme, resulting in vasodilation (FitzGerald & Meisel 2000), by modulating the release of endothelin-1 by endothelial cells (Maes *et al*. 2004) and acting as opioid receptor ligands increasing nitric oxide production which mediates arterial tone (Kris-Etherton *et al*. 2009). An important consideration is the potential impact of a threshold dependency mechanism whereby benefit is conferred in those at low nutrient status, such as calcium, whereas in individuals with adequate baseline status, little effect is observed (McCarron *et al*. 1991; Wennersberg *et al*. 2009; Park & Cifelli 2013). This has important considerations in respect of public health advice on dairy consumption within population groups with different nutritional status. Although the *Rotterdam Study* found an inverse association between low-fat dairy intake and hypertension risk in older adults (Engberink *et al*. 2009), limited evidence exists as to the potential additional benefit of low-fat dairy foods and the type of dairy products in relation to BP reduction. Low-fat dairy is the product of choice in most trials, yet both low- and high-fat alternatives appear to have an overall beneficial effect in relation to BP (Ralston *et al*. 2012). Fumeron *et al*. (2011) reported that consumption of either a variety of dairy products excluding cheese, or cheese alone, and the calcium density of the diet were associated with a lower 9-year diastolic BP after analysing data from the *Epidemiological Study on the Insulin Resistance Syndrome*. Moreover, data from the *Caerphilly Prospective Study* illustrated that when compared to non-milk consumers, men who consumed >586 ml/day had a 10.4 mmHg lower systolic BP after a 22.8-year follow-up (Livingstone *et al*. 2013). Unsurprisingly, greater hypotensive effects of dairy consumption are observed in those with hypertension or who present with calcium sensitivity. In normotensive subjects, dairy consumption is often related to retaining BP homeostasis rather than hypotensive effects (Park & Cifelli 2013).

Elasticity of the blood vessels can be influenced by chronic dietary patterns (Kesse-Guyot *et al*. 2010). Cardiovascular events and all-cause mortality are independently predicted by carotid-femoral pulse wave velocity (PWV), the gold standard measurement of arterial stiffness (Vlachopoulos *et al*. 2010; Van Bortel *et al*. 2012). Further evidence from the *Caerphilly Prospective Study* highlighted that, with the exception of butter consumption, dairy product intake does not impact negatively on PWV (Livingstone *et al*. 2013). Furthermore, augmentation index, another indicator of arterial stiffness, was 1.8% lower in men with the highest quartiles of dairy food consumption (Livingstone *et al*. 2013). Similarly, cross-sectional study findings illustrated that dairy food intake was inversely correlated with PWV (Crichton *et al*. 2012a).

A low-grade systemic inflammation is recognised as a major factor contributing to the development and progression of a number of disorders related to CVD (Labonte *et al*. 2013). Cross-sectional studies that have investigated the relationship between dairy intake and low-grade systemic inflammation have found an inverse association (Salas-Salvado *et al*. 2008; Esmaillzadeh & Azadbakht 2010). However, a review that grouped several studies involving overweight or obese subjects found a degree of heterogeneity which hinders any definite conclusions (Labonte *et al*. 2013). Although there is evidence supporting a beneficial association between dairy consumption and inflammation, the mechanisms are still unclear and studies are either underpowered or use more than one type of dairy product as an intervention, making it difficult to distinguish between dairy products (Labonte *et al*. 2013).

## Dietary strategies for lowering consumption of saturated fat and the implication for cardiovascular disease

### Low-fat milk and dairy products

When compared with low-fat alternatives, there is no established nutritional benefit of whole-fat dairy consumption, except in young children; therefore, the intake of low-fat milk and milk-related products may be considered an effective strategy to lower SFA intake. However, there is currently no consensus on whether fat-reduced dairy foods are associated with a reduced risk of CVD (Benatar *et al*. 2013). Observational studies have indicated that low-fat dairy consumption is an effective strategy to promote lower BP levels (Engberink *et al*. 2009; Toledo *et al*. 2009; van Meijl & Mensink 2011), circulating markers of inflammation (Esmaillzadeh & Azadbakht 2010), the ratio of total cholesterol:HDL-cholesterol (Mensink *et al*. 2003) and LDL-cholesterol concentration (Kai *et al*. 2013), as well as aid in weight maintenance or reduction (Abargouei *et al*. 2012). The *Nurses' Health Study* cohort illustrated that the associated RR of CHD varied according to consumption of high-fat (RR 1.12; 95% CI 1.05–1.20) or low-fat dairy consumption (RR 0.80; 95% CI 0.73–0.87) (Hu *et al*. 1999). On the contrary, findings from a prospective population-based cohort of 33 636 women suggested there were no significant differences between consumption of specific low-fat and high-fat dairy products and MI risk (Patterson *et al*. 2013). Furthermore, findings from a 12-month randomised crossover trial concluded that inclusion of reduced-fat dairy products in the diets of overweight adults had no impact on cardio-metabolic outcomes, including blood lipids, BP and arterial compliance (Crichton *et al*. 2012b). However, before it can be clearly established whether or not removal of milk fat is beneficial to overall cardio-metabolic health, further evidence from well-controlled human intervention studies is required.

### Altering the dairy cow diet to manipulate the fatty acid profile of milk

As an alternative to promoting low-fat dairy product consumption, modification of the fatty acid profile of bovine milk offers a strategy for lowering the population's intake of SFA, by removing SFA from the food chain, while preserving the beneficial contributions that dairy products make to the protein and micronutrient content of the human diet (Shingfield *et al*. 2008). Over 100 studies have explored the potential of partially replacing milk SFA with *cis*-MUFA or *cis*-PUFA through supplementation of the bovine diet with plant oils or oilseeds (Givens & Shingfield 2006; Glasser *et al*. 2008). Through a reduced synthesis of short- and medium-chain SFA by the mammary gland, this feeding strategy enhances the long-chain (>C18) unsaturated fatty acid concentration in the milk (Doreau *et al*. 1999). Inclusion of 49 g/kg of dry matter of rapeseed oil in the ruminant diet for a 28-day period increased *cis*-MUFA from 20 to 33 g/100 g FA, while reducing SFA from 70 to 55–60 g/day FA (Givens *et al*. 2009). Although a more substantial decrease in SFA (∼20 g/100 g of FA) has been documented, the alteration to milk FA composition was adversely linked to voluntary bovine nutrient intake and milk yield compared to the control diet (Givens *et al*. 2003). In order for modification of the composition of ruminant-derived foods to be recognised as a sustainable strategy for reducing SFA intake at a population level, it is essential to find an optimal balance between maximising the unsaturated FA profile of the milk and minimising the impact of the supplementation on animal performance (Givens 2008). Furthermore, it should be considered that ruminal bio-hydrogenation of PUFA results in the formation of intermediates including *trans* 18:1 and leads to small increases in PUFA relative to MUFA concentrations and therefore, it might be more feasible to supplement the bovine diet with MUFA (Shingfield *et al*. 2013).

In addition to the reductions in SFA and increases in *cis*-MUFA, inclusion of unsaturated fatty acids into the bovine-feeding regimen can lead to increased concentrations of naturally produced ruminant *trans* fatty acid (rTFA), namely linoleic acid isomers and *trans* MUFA, in the milk. The intake of *trans* fatty acids (TFA) from industrially hydrogenated vegetable oils is known to have a negative impact on cardiovascular health (Mozaffarian *et al*. 2006; Brouwer *et al*. 2010) and, accordingly, there has been a significant reduction in the level of ‘industrial’ TFA (iTFA) in the food chain (Hulshof *et al*. 1999). Conversely, the association between rTFA and CVD remains inconclusive (Gebauer *et al*. 2011; Brouwer *et al*. 2013) with some studies showing a cardio-protective effect of ruminant sources of *trans* fats (Mozaffarian *et al*. 2006; Jakobsen *et al*. 2008). In an attempt to resolve the conflicting reports, a systematic review and meta-analysis was undertaken by Bendsen *et al*. (2011). They reported that the RR for high vs. low quintiles of total TFA intake (2.8 to approximately 10 g/day) was 1.22 (95% CI 1.08–1.38; *P* = 0.002) for CHD events and 1.24 (95% CI 1.07–1.43; *P* = 0.003) for fatal CHD. rTFA intake (0.5–1.9 g/day) was not significantly associated with CHD risk (RR 0.92; 95% CI 0.76–1.11; *P* = 0.36) although neither was iTFA. There was, however, a trend towards a positive association (RR 1.21; 95% CI 0.97–1.50; *P* = 0.09) for iTFA intake. The authors concluded that while iTFA may be positively related to CHD, rTFA is not, but the limited number of studies available prevented a firm conclusion concerning whether the source of TFA is important. The lack of an association of rTFA with CHD risk may be due to lower intake levels (Bendsen *et al*. 2011). However, at levels currently consumed in the UK diet, there is no evidence of risk from rTFA.

Over the past 10–15 years, the total TFA intake in the UK diet has decreased substantially as a result of voluntary action by the UK food industry; this has led to a greater proportion of the total dietary *trans* fats originating from rTFA (SACN 2007). However, while the proportion of dietary rTFA has increased, the absolute intake of ruminant fat is unchanged. The current dietary intake of TFA in the UK (0.7% of food energy in adults) (DH 2012) is below the recommended population maximum (2% of food energy intake) (DH 1991), with milk and milk products contributing to around 25% of this intake (DH 2011). Consequently, TFA intake from ruminant sources is not seen as a major cause of concern to cardiovascular health at a population level (Tardy *et al*. 2011; Brouwer *et al*. 2013). However, it has yet to be determined whether increasing rTFA intake, through manipulation of the fatty acid profile of milk and dairy products to decrease SFA content, impacts on cardiovascular health (Livingstone *et al*. 2012).

## Impact of modified dairy products on cardiovascular disease risk factors

A review of the current evidence suggests that consumption of modified feed-reduced SFA milk and milk products may be beneficial to CVD risk in healthy and hypercholesterolaemic populations when compared to commercially available whole milk dairy products (Livingstone *et al*. 2012). However, it should be noted that there is a distinct lack of human intervention-based research (Givens 2012; Livingstone *et al*. 2012) and the studies that have been performed have relied on plasma lipid levels as a predictor of CVD risk and on butter as the main test food. Some selected data illustrate that, in comparison to conventional milk, cheese, butter and ice cream (70 g/100 g SFA, 28 g/100 g *cis*-MUFA), total cholesterol and LDL-cholesterol were significantly lowered following a 3-week period of consuming matched fat-modified dairy products (51 g/100 g SFA, 39 g/100 g *cis*-MUFA) (Noakes *et al*. 1996). Dairy products are complex, nutrient-dense foods and focusing on a single outcome measure could lead to misleading conclusions by failing to establish the impact on other CVD risk factors.

Evidence suggests that some of the effects of SFA on CVD risk are mediated by impairment in endothelial function and subsequent establishment of atherosclerosis (Nicholls *et al*. 2006; Blumenthal *et al*. 2010; Vafeiadou *et al*. 2012) and by influencing postprandial lipaemia (Berry & Sanders 2005). Endothelial dysfunction, an early modifiable event in the coronary atherosclerotic process, is positively associated with increased risk of CVD (De Caterina 2000; Schachinger *et al*. 2000). Flow-mediated dilation, which measures the vasodilatory response of the brachial artery to an increase in blood flow-associated shear stress and carotid intima-media thickness, can be used to non-invasively assess endothelial function and arterial structural changes, respectively (Anderson 2006). As previously discussed, it is recommended that arterial stiffness, a surrogate marker of central arterial function, should also be evaluated when exploring the impact of modified dairy consumption on cardiovascular risk factors (Givens 2012). Postprandial lipaemia, characterised by elevated and prolonged triacylglycerol concentrations in the fed state, is influenced by the type and quantity of the meal fat (Chong *et al*. 2010; Jackson & Lovegrove 2012; Jackson *et al*. 2012). Postprandial lipaemia is a significant independent risk marker of CVD that requires attention in future modified fat studies (Nordestgaard *et al*. 2007). Further research is warranted to examine the impact of fat-modified or any total low-fat or full-fat dairy consumption on holistic measures of CVD including vascular function, arterial stiffness, postprandial lipaemia and inflammation.

This is currently being addressed at the University of Reading in the *RESET* (*REplacement of SaturatEd fat in dairy on Total cholesterol*) *Study* (ClinicalTrials.gov NCT02089035), a 3-year Medical Research Council funded project that is investigating the impact of reducing SFA intake by using modified milk and dairy products on vascular function and CVD risk biomarkers, without limiting dairy product consumption. This will be achieved by producing milk and dairy products that have a substantial proportion of the SFA replaced with *cis*-MUFA. In a randomised, crossover, double-blind, controlled study, it will be determined whether modified dairy product consumption improves vascular function and other CVD risk biomarkers relative to typical commercially available products in both acute and chronic settings. The project, which started in late 2013, will provide unique evidence to inform public health policy on food-based dietary recommendations for CVD risk reduction.

## Conclusion

Much of the UK population currently exceeds the dietary SFA recommendation of <11% of food energy intake, with milk and dairy products as a group making a considerable contribution to SFA intake in the average UK diet. While it is intuitive to consider further reducing dairy consumption as a means of decreasing SFA intake, epidemiological data suggest that this strategy may be counterproductive, given the array of cardio-metabolic benefits that milk products appear to offer to human health. Fat-reduced or fat-modified dairy product consumption may offer a more feasible option for reducing intake of SFA with minimal change to habitual eating patterns and, hence, CVD risk at a population level. Nevertheless, before the impact of both fat-reduced and modified dairy consumption on CVD risk reduction can be evaluated, it is essential to conduct more robust, controlled human intervention-based research using both traditional and novel assessments of cardiovascular risk. Additionally, more research is required to differentiate between dairy food matrices.
